# Cataract subtype risk factors identified from the Korea National Health and Nutrition Examination survey 2008–2010

**DOI:** 10.1186/1471-2415-14-4

**Published:** 2014-01-10

**Authors:** Tyler Hyung Taek Rim, Min-hyung Kim, Woon Cho Kim, Tae-Im Kim, Eung Kweon Kim

**Affiliations:** 1 Department of Ophthalmology, Yonsei University College of Medicine, 50 Yonsei-ro, Seodaemun-gu, Seoul, Korea; 2 Seoul National University College of Medicine, Seoul, Republic of Korea; 3 Hwaseong City Health Center, Hwaseong-si, Gyeonggi-do, Korea; 4 Emory University School of Medicine, 30322 Atlanta, Georgia, USA; 5 Corneal Dystrophy Research Institute, Yonsei University College of Medicine, Seoul, Korea; 6 Severance Biomedical Science Institute, Yonsei University College of Medicine, Seoul, Korea; 7 Brain Korea 21 Project for Medical Science, Yonsei University, Seoul, Korea

**Keywords:** Cataract, Cataract subtype, Cataract risk factors, KNHANES

## Abstract

**Background:**

To assess the socio-demographic and health-related risk factors associated with cataract subtypes in Korea.

**Methods:**

A total of 11,591 participants (aged ≥40 years) were selected from the Korean National Health and Nutrition Examination Survey between 2008 and 2010. The Korean Ophthalmologic Society conducted detailed ophthalmologic examinations on these participants based on the Lens Opacity Classification System III. Risk factors for developing any type of cataract, and its subtypes (nuclear, cortical, posterior subcapsular and mixed), were identified from univariate and multivariate logistic regression analysis.

**Results:**

The prevalence of cataracts was 40.1% (95% CI, 37.8 − 42.3%) in participants over 40 years old. Older age, lower monthly household income, lower education, hypercholesterolemia, hypertension, and diabetes mellitus (DM) were independent risk factors for development of any cataract. Older age, lower monthly household income, lower education, hypercholesterolemia, and DM were independent risk factors for development of pure cortical cataracts. Older age, lower education, metabolic syndrome, and DM were independent risk factors for development of pure nuclear cataracts. Older age and DM were independent risk factors for development of pure posterior subcapsular cataracts. Older age, lower monthly household income, lower education, and DM were independent risk factors for development of mixed cataracts.

**Conclusion:**

Although socioeconomic disparities are related to cataract development, this study identified several “modifiable” risk factors that may help to lower the incidence of cataracts and associated vision loss. Improved control of blood pressure, blood, glucose, and cholesterol may help to reduce the incidence of cataracts in the general Korean population.

## Background

**Figure 1:**
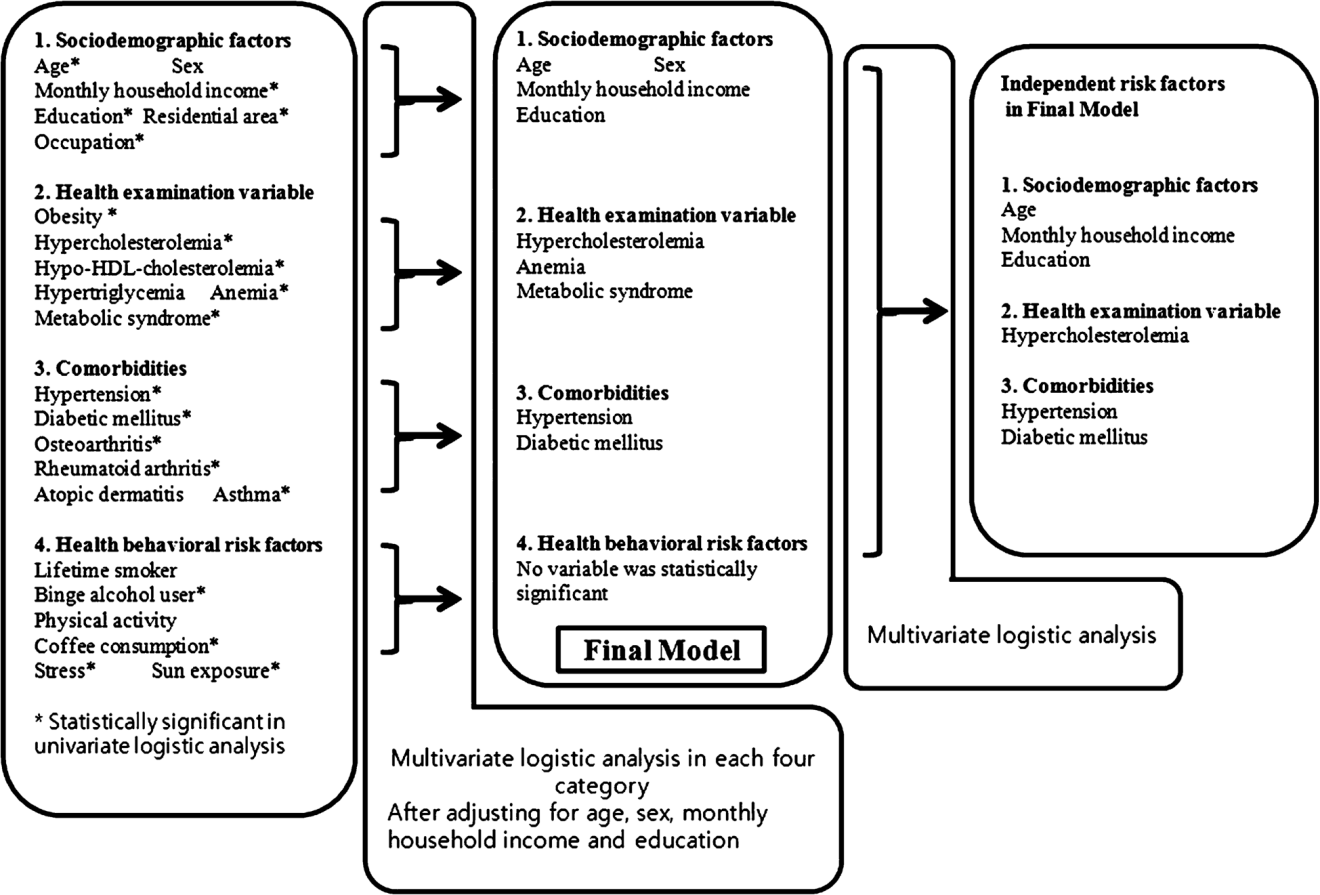
**Study framework flowchart outlining a stepwise approach to identifying risk factors for cataracts, as analyzed with univariate and multivariate analyses.** The independent variables were divided into four categories: Socio-demographic factors; Health examination variables based on blood tests and physical examinations; Comorbidities; and Health behavioral risk factors based on direct interviews.

Age-related cataracts are the leading cause of blindness worldwide [1]. Identifying cataract risk factors can lead to various prevention and treatment options that will ultimately lessen the economic and public health burden of this disease. Previous studies have evaluated some of the modifiable and non-modifiable risk factors for cataracts, including educational status [1,2], smoking [3], diabetes [4,5], sunlight exposure [6,7], body mass index [8-10], steroid drug use [11], asthma [12], and estrogen replacement therapy [13-15]. Although these studies detail some of the pathophysiology of this multi-factorial disease in Western countries, risk factors specific to Asian populations are not well known. In fact, most studies that evaluated risk factors for specific cataract types (e.g., nuclear, cortical, and posterior subcapsular opacity [PSCO]) were performed in western countries [16-18]. A small but growing number of studies on specific cataract types in Asian countries have recently been performed in Japan, Taiwan, Singapore, and China [19-22]. The Korea National Health and Nutrition Examination Survey (KNHANES) is a nationally representative survey conducted by the Ministry of Health and Welfare that provides data on vision status, healthcare use, and other socio-demographic factors. KNHANES results and statistics are readily available at http://knhanes.cdc.go.kr. Our objective, therefore, was to use ophthalmologic examination results from KNHANES to investigate the socio-demographic and health-related risk factors associated with cataract subtypes (Figure 1).

## Methods

### Study design and population

We have previously described our sampling, enumeration, visual acuity, and ocular examination procedures [23,24]. The Korea Center for Disease Control and Prevention (KCDCP) conducted a KNHANES series (I, II, and III) in 1998, 2001, and 2005, to examine general health and nutritional status of Koreans. For KNHANES IV (2007–2009), however, the survey became an annual rolling survey that used a stratified, multi-stage, clustered sampling method (based on 2005 National Census data) to randomly select 24,871 individuals across 500 national districts that represented the civilian, non-institutionalized South Korean population. KNHANES V (2010–2012) also randomly sampled households but across 576 national districts (192 enrolled each year). These households were also selected with a stratified, multi-stage clustered sampling method but were based on 2009 National Resident demographics. Surveys prior to KNHANES IV were able to be analyzed and could be considered a national representative sample after 3 years when the survey was completed, but rolling survey sampling methods were applied from KNHANES IV that allowed annual analysis of nationally representative data.

**Figure 2:**
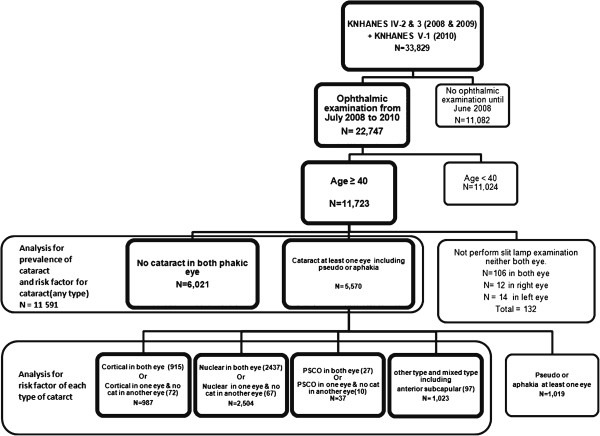
Flow diagrams showing selection of the study population.

The KNHANES is divided into three parts: the Health Interview Survey, the Health Examination Survey, and the Nutrition Survey. Because the Korean Ophthalmologic Society participated in this survey after July 2008, ophthalmologic interviews and examinations were also conducted with the same participants. All members of each selected household were asked to participate in the survey, with a participation rate of 82.0%. We omitted participants less than 40 years old who had incomplete slit-lamp examinations, leaving a total of 11,591 participants from 2008 to 2010 (Figure 2). This survey was reviewed and approved by the Institutional Review Board of the Korea Centers for Disease Control and Prevention, and all participants provided written informed consent following the Declaration of Helsinki.

### Ophthalmologic examinations

**Figure 3:**
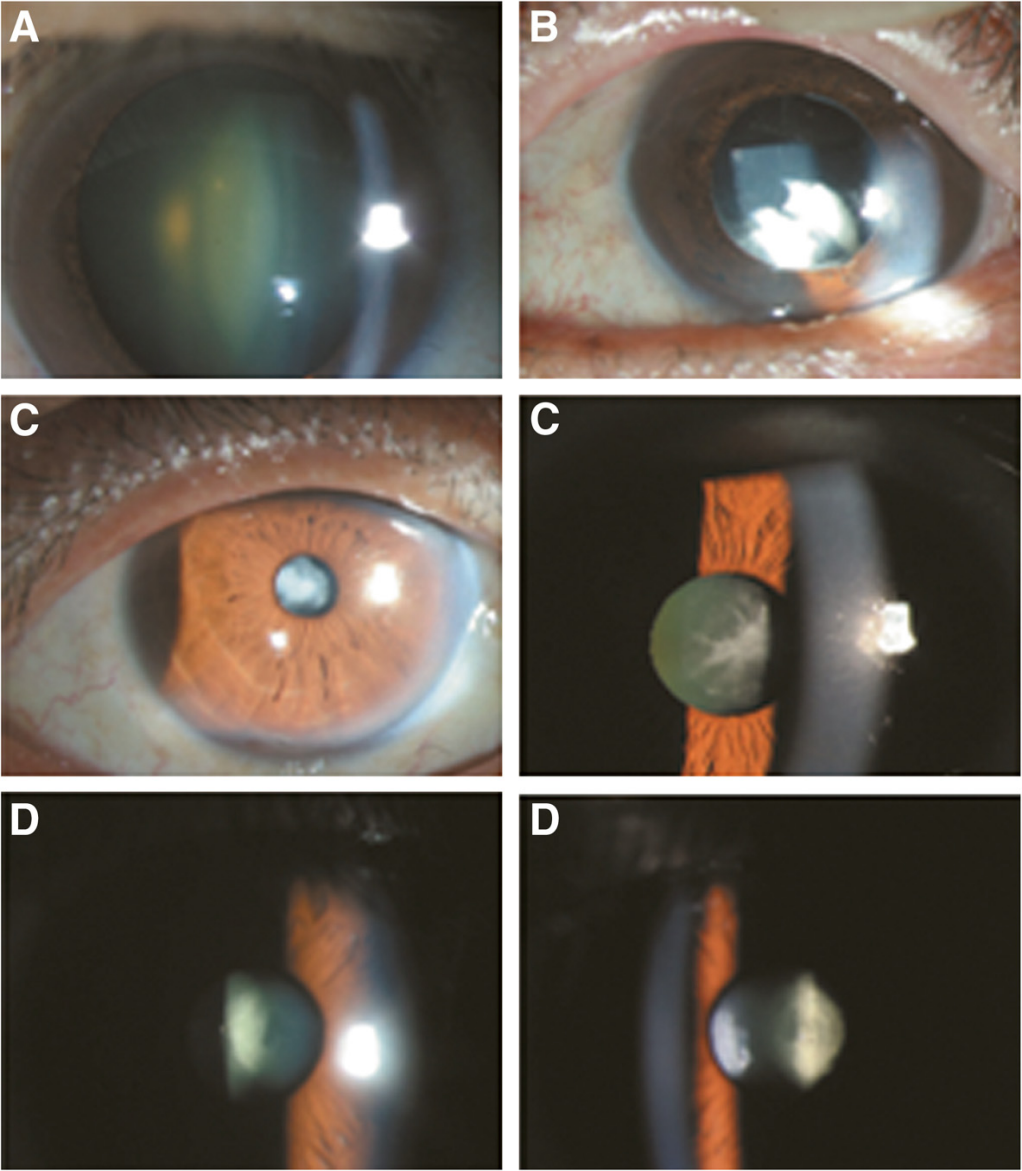
**Standard pictures for each subtype. A.** Nuclear type, **B.** Cortical type, **C.** Anterior capsular type, **D.** Posterior capsular type.

Designated ophthalmologists performed a structured slit-lamp examination (Haag-Streit model BQ-900, Haag-Streit AG, Koeniz, Switzerland) to determine disease occurrence in the anterior segment of the eye (e.g., pterygium and cataract). Examinees were seated in the examination chair, resting their chin and forehead on the support. An illuminator was positioned behind the examinees’ ears; the angle between the illuminator and the microscope was 30 ~ 45 degrees with a 10× magnification.Without iridodilator usage, the characteristics of lens were assessed using slit lamp with proper brightness, height, and width. The overall characteristics of the lens were examined with a wider slit lamp, and the type and severity of the cataract was determined according to transparency, turbidity, pigments, vacuoles and nuclei. Each layer of the lens was examined with the focused slit lamp from the anterior capsule to the posterior capsule. Aphakia and pseudophakia were recorded separately, and excluded from the subtype analysis. The type of cataract was categorized according to Lens Opacity Classification System III (LOCS III) grading in both eyes, as nuclear, cortical, PSCO, or mixed (including anterior subcapsular). Standard pictures for each subtype were provided for each examiner (Figure 3). The quality of the survey was verified by the Epidemiologic Survey Committee of the Korean Ophthalmologic Society. Training of participating residents was periodically performed by acting staff members of the National Epidemiologic Survey Committee of the Korean Ophthalmologic Society.

### Outcome variable

**Figure 4:**
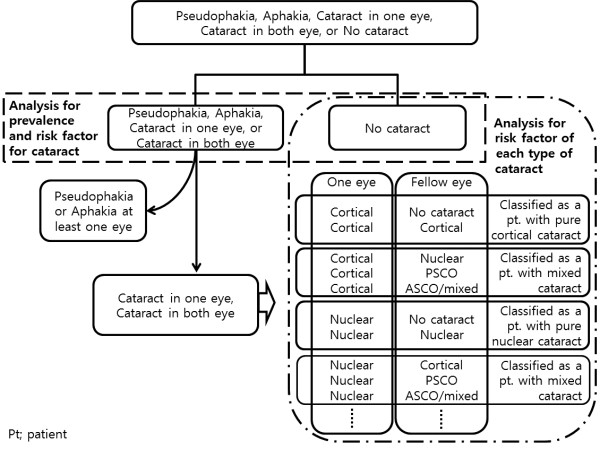
Flow chart shows how to classify the each subtype of cataract in detail.

To identify risk factors for any type of cataract, we first verified cataract occurrence in a person with the presence of a nuclear, cortical, anterior subcapsular, or posterior subcapsular cataract in at least one eye. For statistical purposes, we also included pseudophakic and aphakic eyes as operated cataracts for calculating prevalence. To analyze and evaluate risk factors for each type of cataract, we defined the cataract subtypes as follows. Participants with no type of cataract in either phakic eye were defined as having no cataract. Individuals who had a cortical cataract in at least one eye were defined as having a pure cortical cataract. Individuals with either a nuclear cataract or a PSCO were similarly defined. Individuals with a mixed type cataract, which included an anterior subcapsular type in at least one eye, were defined as having a mixed type cataract (Figure 2). Figure 4 shows how the cataract subtypes were categorized in detail using a flow chart.

### Independent variables

The independent variables were divided into four categories: (1) socio-demographic factors, (2) health examination variables, (3) comorbidities, and (4) health behavioral risk factors. The income per adult equivalent was calculated with the following formula: household income divided by the square root of the number of people in the household [25]. Binge alcohol users were defined as either men who consumed more than seven drinks on a single occasion or women who consumed more than five drinks on a single occasion, both at a prevalence of at least once per month [26]. Respondents who reported that they were current smokers and had smoked at least 100 cigarettes in their lifetime were considered lifetime smokers [27]. We used the World Health Organization BMI-defined obesity standard (international standard) to define both obesity and underweight (≥25 kg/m2 and <18 kg/m2, respectively) for adults. Hypercholesterolemia was defined for any of the following three cases: 1) a total cholesterol level >240 mg/dL from a blood test taken after fasting, 2) the use of lipid-lowering drugs, or 3) diagnosis of dyslipidemia by a physician. HDL-cholesterol levels <40 mg/dL were defined as hypo-HDL-cholesterolemia and triglyceride levels >200 mg/dL were defined as hypertriglycemia. In this study, subjects who fulfilled at least three of the following five components were defined as exhibiting metabolic syndrome: 1) central obesity (waist circumference: ≥90 cm for Korean men, ≥85 cm for Korean women; The Korean Society for the Study of Obesity proposed 90 cm and 85 cm as the appropriate abdominal circumferences for obesity consideration in Korean men and women, respectively [28,29]), 2) hypertriglyceridemia (≥150 mg/dL), 3) low HDL cholesterol (men <40 mg/dL, women <50 mg/dL), 4) high blood pressure (systolic blood pressure ≥130 mmHg, diastolic blood pressure ≥85 mmHg, or receiving hypertension drug treatment) and 5) hyperglycemia (fasting serum glucose ≥100 mg/dL).

### Statistical methods

We report descriptive statistics for each response. We determined age-specific prevalence of cataracts in Koreans with weighting recommended in KNHANES IV and V-1. To weight KNHANES IV in accordance to guidelines in the 2005 Census of Korea, we performed a post-stratification adjustment to response and extraction rates that included the same distribution of the 2005 Korean population, according to sex and age groups and at 5-year intervals. Finally, the sum of the weight of KNHANES IV was considered equal to that in the Korean population as of 2005. We weighted KNHANES V-1 in a similar manner but based it on the 2010 Korean population and in accordance with the 2010 Census of Korea.

We used a three-step, multi-dimensional approach to identify cataract risk factors. First, we calculated unadjusted odds ratios and 95% CIs with univariate logistic regression analysis. Second, we applied multivariate logistic regression analysis on all variables in each category, after adjusting for age, sex, monthly household income, and education. Finally, we used multivariate logistic regression analysis to determine independent risk factors. All risk factors that were identified by multivariate analysis were included in the final multivariate analysis (Final Model; middle column in Figure 2). All statistical tests were two-sided and performed with Stata/SE 12.1 software (StataCorp, College Station, TX, USA).

## Results

### Baseline characteristics of the study population

The mean (± standard error) age of the final 11,591 participants was 58.4 ± 0.1 years. Of those participants, 43.1% were men, 70.1% were living in an urban area, 14.6% had hypercholesterolemia, 9.9% had anemia, 41.5% had metabolic syndrome, 30.0% had hypertension, and 10.9% had diabetes mellitus (DM). Table 1 lists the detailed baseline characteristics of the study population.

**Table 1:** Characteristics of the study population (N = 11,591)

	**n**	**%**
**1. Socio-demographic factors**		
Age (years)		
40–49	3353	28.9
50–59	3019	26.1
60–69	2813	24.3
70–80	1959	16.9
80+	447	3.9
Sex		
Men	5000	43.1
Women	6591	56.9
Monthly household income		
Lowest quintile	3108	26.8
2nd to 4th quintile	5946	51.3
Highest quintile	2355	20.3
Education		
Elementary school	4644	40.1
Middle school	1779	15.4
High school	3111	26.8
University or higher	1929	16.6
Residential area		
Urban	8124	70.1
Rural	3467	29.9
Occupation		
Administrator, management, and professional	883	7.6
Business and financial operations	548	4.7
Sales and related	1416	12.2
Farming, fishing, and forestry	1539	13.3
Installation, maintenance, and repair	1089	9.4
Laborer	1208	10.4
Unemployed	4761	41.1
**2. Health examination variable**		
Obesity		
Underweight	7223	62.3
Normal	3980	34.3
Obesity	357	3.1
Hypercholesterolemia		
No	8978	77.5
Yes	1695	14.6
Hypo-HDL-cholesterolemia		
No	7613	65.7
Yes	3122	26.9
Hypertriglycemia		
No	7257	62.6
Yes	1547	13.4
Anemia		
No	9778	84.4
Yes	1150	9.9
Metabolic syndrome		
No	6782	58.5
Yes	4809	41.5
**3. Comorbidities**		
Hypertension		
No	8033	69.3
Yes	3472	30.0
Diabetic mellitus		
No	10237	88.3
Yes	1267	10.9
Osteoarthritis		
No	9596	82.8
Yes	1907	16.5
Rheumatoid arthritis		
No	11161	96.3
Yes	342	3.0
Atopic dermatitis		
*No*	11339	97.8
Yes	163	1.4
Asthma		
No	11083	95.6
Yes	421	3.6
**4. Health behavioral risk factors**		
Lifetime smoker		
No	6797	58.6
Yes	4704	40.6
Binge alcohol user		
No	8375	72.3
Yes	3140	27.1
Physical activity of moderate intensity		
never	11078	95.6
more than once in a week	394	3.4
Coffee consumption		
Never	1393	12.0
1–6 cups per week	2236	19.3
≥7 cups per week	6476	55.9
Stress		
Least stressful	2249	19.4
Moderately stressful	8671	74.8
Extremely stressful	557	4.8
Sun exposure		
<5 hr/day	8182	70.6
≥5 hr/day	3286	28.4

### Cataract prevalence

Table 2 provides the prevalence of cataract specific to age and sex. The listed data are percentages of prevalence and the 95% confidence interval (CI). The overall prevalence of cataracts in subjects aged 40 years and older was 40.1% (95% CI, 37.8–42.3%). The prevalence for each type of cataract were 7.4% (95% CI, 6.4–8.5%) for pure cortical type, 20.3% (95% CI, 18.2–22.3%) for pure nuclear type, 0.3% (95% CI, 0.2–0.5%) for pure posterior subcapsular type, and 7.5% (95% CI, 6.6–8.4%) for mixed type.

**Table 2:** The prevalence of cataract

	**%**	**95% CI**
**All**		
40 ~ 49	10.4	(8.4 −12.4 )
50 ~ 59	33.1	(29.6 −36.6 )
60 ~ 69	69.4	(66.0 −72.8 )
70 ~ 80	91.2	(89.2 −93.3 )
80~	97.9	(96.4 −99.5 )
≥40*	40.1	(37.9 −42.3 )
≥50	58.1	(55.5 −60.8 )
≥60	80.2	(77.9 −82.4 )
≥70	92.7	(91.0 −94.3 )
≥80	97.9	(96.4 −99.5 )
**Men**		
40 ~ 49	10.7	(8.5 −13.0 )
50 ~ 59	34.6	(30.4 −38.8 )
60 ~ 69	68.4	(64.2 −72.7 )
70 ~ 80	89.6	(86.6 −92.5 )
80~	96.7	(93.7 −99.7 )
**Women**		
40 ~ 49	10.0	(7.7 −12.3 )
50 ~ 59	31.6	(27.8 −35.4 )
60 ~ 69	70.3	(66.5 −74.0 )
70 ~ 80	92.3	(90.0 −94.6 )
80~	98.5	(97.0 −100.0 )

* ≥ X = subjects aged X years and older.

### Factors associated with cataracts

The factors from the univariate analysis that were significantly associated with cataracts (see Table 3 for odd ratios and 95% CIs) included all variables except sex, hypertriglycemia, atopic dermatitis, lifetime smoking, and physical activity (left column in Figure 1). In the multivariate analysis of all socio-demographic factors (Table 4), four risk factors were statistically significant: age, sex, monthly household income and education. Participants with hypercholesterolemia, anemia, and metabolic syndrome were more likely to have cataracts in their health examination variables, after adjusting for the above four significant socio-demographic factors. For comorbidities, participants with either hypertension or DM were more likely to have cataracts after adjusting for age, sex, monthly household income, and education. For the multivariate regression analysis, none of the health behavioral risk variables were significantly related to cataract occurrence.

**Table 3:** Factors associated with risk of developing cataracts - univariate analysis (N = 11,591)

	**Univariate OR**	**95% CI**	**p**
**1. Socio-demographic factors**			
Age (years)			
40–49	1.0 (ref)		
50–59	**4.3**	**3.8–5.0**	**<0.01**
60–69	**21.0**	**18.3–24.1**	**<0.01**
70–80	**92.6**	**76.4–112.4**	**<0.01**
80+	**344.5**	**187.4–633.1**	**<0.01**
Sex			
Men	1.0 (ref)		
Women	**1.0**	**1.0–1.1**	**0.35**
Monthly household income			
Lowest quintile	1.0 (ref)		
2nd to 4th quintile	**0.2**	**0.2–0.3**	**<0.01**
Highest quintile	**0.1**	**0.1–0.1**	**<0.01**
Education			
Elementary school	1.0 (ref)		
Middle school	**0.3**	**0.3–0.4**	**<0.01**
High school	**0.2**	**0.2–0.2**	**<0.01**
University or higher	**0.1**	**0.1–0.1**	**<0.01**
Residential area			
Urban	1.0 (ref)		
Rural	**1.8**	**1.6–1.9**	**<0.01**
Occupation			
Administrator, management, and professional	1.0 (ref)		
Business and financial operations	**0.9**	**0.7–1.2**	**0.49**
Sales and related	**1.6**	**1.3–1.9**	**<0.01**
Farming, fishing, and forestry	**6.8**	**5.6–8.3**	**<0.01**
Installation, maintenance, and repair	**1.6**	**1.3–2.0**	**<0.01**
Laborer	**3.6**	**2.9–4.4**	**<0.01**
Unemployed	**6.5**	**5.5–7.8**	**<0.01**
**2. Health examination variable**			
Obesity			
Underweight	1.0 (ref)		
Normal	**1.0**	**0.9–1.1**	**0.80**
Obesity	**2.1**	**1.7–2.7**	**<0.01**
Hypercholesterolemia			
No	1.0 (ref)		
Yes	**1.5**	**1.3–1.6**	**<0.01**
Hypo-HDL-cholesterolemia			
No	1.0 (ref)		
Yes	**1.4**	**1.2–1.5**	**<0.01**
Hypertriglycemia			
No	1.0 (ref)		
Yes	**1.1**	**1.0–1.2**	**0.13**
Anemia			
No	1.0 (ref)		
Yes	**1.3**	**1.2–1.5**	**<0.01**
Metabolic syndrome			
No	1.0 (ref)		
Yes	**2.1**	**1.9–2.2**	**<0.01**
**3. Comorbidities**			
Hypertension			
No	1.0 (ref)		
Yes	**3.1**	**2.8–3.3**	**<0.01**
Diabetic mellitus			
No	1.0 (ref)		
Yes	**3.1**	**2.7–3.5**	**<0.01**
Osteoarthritis			
No	1.0 (ref)		
Yes	**2.8**	**2.5–3.1**	**<0.01**
Rheumatoid arthritis			
No	1.0 (ref)		
Yes	**2.1**	**1.7–2.6**	**<0.01**
Atopic dermatitis			
No	1.0 (ref)		
Yes	**0.7**	**0.5–1.0**	**0.06**
Asthma			
No	1.0 (ref)		
Yes	**1.9**	**1.6–2.3**	**<0.01**
**4. Health behavioral risk factors**			
Lifetime smoker			
No	1.0 (ref)		
Yes	**1.0**	**0.9–1.1**	**0.82**
Binge alcohol user			
No	1.0 (ref)		
Yes	**0.5**	**0.4–0.5**	**<0.01**
Physical activity of moderate intensity			
Never	1.0 (ref)		
>5 times per week	**1.1**	**0.9–1.3**	**0.51**
Coffee consumption			
Never	1.0 (ref)		
1–6 cups per week	**0.7**	**0.6–0.8**	**<0.01**
≥7 cups per week	**0.5**	**0.4–0.5**	**<0.01**
Stress			
Least stress	1.0 (ref)		
Moderately stressful	**0.4**	**0.4–0.5**	**<0.01**
Extreme stress	**0.6**	**0.5–0.7**	**<0.01**
Sun exposure			
<5 hr/day	1.0 (ref)		
≥5 hr/day	**1.7**	**1.6–1.9**	**<0.01**

**Table 4:** Factors associated with risk of developing cataracts after adjusting for significant socio-demographic factors* - multivariate analysis for each variable cluster (N = 11,591)

	**Multivariate OR**	**95% CI**	**p**
**1. Socio-demographic factors**			
Age (years)			
40–49	**1.0 (ref)**		
50–59	**3.7**	**3.2–4.3**	**<0.01**
60–69	**14.9**	**12.7–17.4**	**<0.01**
70–80	**57.3**	**46.0–71.3**	**<0.01**
80+	**192.7**	**103.6–358.4**	**<0.01**
Sex			
Men	**1.0 (ref)**		
Women	**0.9**	**0.8–1.0**	**0.02**
Monthly household income			
Lowest quintile	**1.0 (ref)**		
2nd to 4th quintile	**0.9**	**0.7–1.0**	**0.04**
Highest quintile	**0.7**	**0.6–0.9**	**<0.01**
Education			
Elementary school	**1.0 (ref)**		
Middle school	**0.8**	**0.7–0.9**	**<0.01**
High school	**0.7**	**0.6–0.8**	**<0.01**
University or higher	**0.6**	**0.5–0.7**	**<0.01**
Residential area			
Urban	1.0 (ref)		
Rural	1.0	0.9–1.2	0.57
Occupation			
Administrator, management, and professional	1.0 (ref)		
Business and financial operations	1.0	0.8–1.4	0.78
Sales and related	1.0	0.8–1.3	0.79
Farming, fishing, and forestry	1.1	0.8–1.4	0.49
Installation, maintenance, and repair	1.0	0.7–1.3	0.83
Laborer	1.1	0.8–1.4	0.54
Unemployed	1.2	1.0–1.5	0.09
**2. Health examination variable**			
Obesity			
Underweight	1.0 (ref)		
Normal	1.0	0.8–1.1	0.44
Obesity	1.4	0.9–1.9	0.10
Hypercholesterolemia			
No	1.0 (ref)		
Yes	**1.2**	**1.0–1.4**	**0.03**
Hypo-HDL-cholesterolemia			
No	1.0 (ref)		
Yes	1.0	0.8–1.1	0.64
Hypertriglycemia			
No	1.0 (ref)		
Yes	0.9	0.7–1.0	0.10
Anemia			
No	1.0 (ref)		
Yes	**1.2**	**1.0–1.5**	**0.05**
Metabolic syndrome			
No	1.0 (ref)		
Yes	**1.2**	**1.1–1.4**	**<0.01**
**3. Comorbidities**			
Hypertension			
No	1.0 (ref)		
Yes	**1.2**	**1.0–1.3**	**<0.01**
Diabetic mellitus			
No	1.0 (ref)		
Yes	**1.7**	**1.4–2.0**	**<0.01**
Osteoarthritis			
No	1.0 (ref)		
Yes	1.1	0.9–1.2	0.31
Rheumatoid arthritis			
No	1.0 (ref)		
Yes	1.1	0.8–1.4	0.55
Atopic dermatitis			
No	1.0 (ref)		
Yes	0.9	0.6–1.3	0.45
Asthma			
No	1.0 (ref)		
Yes	1.0	0.8–1.3	0.92
**4. Health behavioral risk factors**			
Lifetime smoker			
No	1.0 (ref)		
Yes	1.0	0.9–1.2	0.59
Binge alcohol user			
No	1.0 (ref)		
Yes	0.9	0.8–1.0	0.14
Physical activity of moderate intensity			
Never	1.0 (ref)		
>5 times per week	1.0	0.8–1.3	0.93
Coffee consumption			
Never	1.0 (ref)		
1–6 cups per week	0.9	0.8–1.1	0.50
≥7 cups per week	0.9	0.7–1.0	0.09
Stress			
Least stress	1.0 (ref)		
Moderate stress	0.9	0.8–1.0	0.07
Extreme stress	1.1	0.8–1.4	0.59
Sun exposure			
<5 hr/day	1.0 (ref)		
≥5 hr/day	1.1	1.0–1.2	0.09

Significant risk factors for cataract were combined into a final model in Figure 1. In the multivariate analysis for cataract based on the final model (Table 5), three risk factors were statistically significant among socio-demographic variables: (1) age [age 40–49 = 1.0 (ref), adjusted odds ratio (aOR) of age 50–59 = 3.5 (95% CI, 3.0–4.1), aOR of age 60–69 = 14.3 (95% CI, 12.1-16.8), aOR of age 70–80 = 53.1 (95% CI, 42.5-66.4), and aOR of age 80 + = 194.1 (95% CI, 94.5-398.6)]; (2) monthly household income [1.0(ref) in the lowest quintile, aOR of the 2nd to 4th quintile = 0.9 (95% CI, 0.7–1.0), and aOR of the highest quintile = 0.7 (95% CI, 0.6–0.9)]; and (3) education [1.0 (ref) in elementary school, aOR of middle school = 0.8 (95% CI, 0.7–0.9), aOR of high school = 0.7 (95% CI, 0.6–0.8), and aOR of either university or higher = 0.6 (95% CI, 0.5–0.7)]. Participants with hypercholesterolemia (aOR = 1.2; 95% CI, 1.0–1.3) were more likely to have cataracts in their health examination variables. For comorbidities, participants with hypertension (aOR = 1.1; 95% CI, 1.0–1.3) or DM (aOR = 1.6; 95% CI, 1.3–1.9) were more likely to have cataracts. In the multivariate analysis for cataract subtypes (Table 6), five factors were statistically significant for the pure cortical cataract type: age, monthly household income, education, hypercholesterolemia, and DM. For the pure nuclear type of cataract, four factors were statistically significant: age, education, metabolic syndrome, and DM. For the pure posterior subcapsular opacity, two factors were statistically significant: age and DM. Finally, for the mixed type of cataract, four factors were statistically significant: age, monthly household income, education, and DM.

**Table 5:** Factors associated with risk of cataract development - Final model (n = 11,591)

	**Univariate OR**	**Multivariate OR**	**95% CI**	**p**
**1. Socio-demographic factors**				
Age (years)				
40–49	1.0 (ref)	**1.0 (ref)**		
50–59	4.3	**3.5**	**3.0–4.1**	**<0.01**
60–69	21.0	**14.3**	**12.1–16.8**	**<0.01**
70–80	92.6	**53.1**	**42.5–66.4**	**<0.01**
80+	344.5	**194.1**	**94.5–398.6**	**<0.01**
Sex				
Men	1.0 (ref)	1.0 (ref)		
Women	1.0	0.9	0.8–1.0	0.14
Monthly household income				
Lowest quintile	1.0 (ref)	**1.0 (ref)**		
2nd to 4th quintile	0.2	**0.9**	**0.7–1.0**	**0.05**
Highest quintile	0.1	**0.7**	**0.6–0.9**	**<0.01**
Education				
Elementary school	1.0 (ref)	**1.0 (ref)**		
Middle school	0.3	**0.8**	**0.7–0.9**	**<0.01**
High school	0.2	**0.7**	**0.6–0.8**	**<0.01**
University or higher	0.1	**0.6**	**0.5–0.7**	**<0.01**
**2. Health examination variable**				
Hypercholesterolemia				
No	1.0 (ref)	**1.0 (ref)**		
Yes	1.5	**1.2**	**1.0–1.3**	**0.02**
Anemia				
No	1.0 (ref)	1.0 (ref)		
Yes	1.3	1.1	0.9–1.3	0.18
Metabolic syndrome				
No	1.0 (ref)	1.0 (ref)		
Yes	2.1	1.1	0.9–1.2	0.32
**3. Comorbidities**				
Hypertension				
No	1.0 (ref)	**1.0 (ref)**		
Yes	3.1	**1.1**	**1.0–1.3**	**0.02**
Diabetic mellitus				
No	1.0 (ref)	**1.0 (ref)**		
Yes	3.1	**1.6**	**1.3–1.9**	**<0.01**

**Table 6:** Factors associated with risks of developing subtypes of cataracts - Final model (N = 11,591)

	**Pure Cortical type n = 7008**		**Pure Nuclear type**		**Pure PSCO type**		**Mixed type**	
	**Multivariate OR**	**p value**	**Multivariate OR**	**p value**	**Multivariate OR**	**p value**	**Multivariate OR**	**p value**
**1. Socio-demographic factors**								
Age (years)								
40–49	1.0 (ref)		1.0 (ref)		1.0 (ref)		1.0 (ref)	
50–59	**2.8**	**<0.01**	**4.1**	**<0.01**	**3.2**	**0.07**	**4.1**	**<0.01**
60–69	**9.0**	**<0.01**	**15.7**	**<0.01**	**6.9**	**<0.01**	**22.6**	**<0.01**
70–80	**26.4**	**<0.01**	**44.8**	**<0.01**	**24.1**	**<0.01**	**104.3**	**<0.01**
80+	**59.6**	**<0.01**	**138.2**	**<0.01**	**135.9**	**<0.01**	**414.0**	**<0.01**
Sex								
Men	1.0 (ref)		1.0 (ref)		1.0 (ref)		1.0 (ref)	
Women	0.9	0.07	1.0	0.87	1.0	0.92	1.1	0.50
Monthly household income								
Lowest quintile	1.0 (ref)		1.0 (ref)		1.0 (ref)		1.0 (ref)	
2nd to 4th quintile	**0.7**	**<0.01**	1.0	0.97	0.5	0.07	**0.8**	**0.02**
Highest quintile	**0.7**	**<0.01**	1.0	0.78	0.4	0.12	**0.6**	**<0.01**
Education								
Elementary school	1.0 (ref)		1.0 (ref)		1.0 (ref)		1.0 (ref)	
Middle school	0.8	0.11	**0.8**	**0.02**	0.7	0.52	0.8	0.11
High school	**0.6**	**<0.01**	**0.8**	**0.02**	1.0	0.93	**0.6**	**<0.01**
Univ. or higher	**0.5**	**<0.01**	**0.6**	**<0.01**	0.9	0.93	0.9	0.56
**2. Health examination variable**								
Hypercholesterolemia								
No	1.0 (ref)		1.0 (ref)		1.0 (ref)		1.0 (ref)	
Yes	**1.3**	**0.02**	1.0	0.61	1.6	0.28	1.1	0.26
Anemia								
No	1.0 (ref)		1.0 (ref)		1.0 (ref)		1.0 (ref)	
Yes	0.9	0.70	1.1	0.29	1.0	0.96	1.3	0.12
Metabolic syndrome								
No	1.0 (ref)		1.0 (ref)		1.0 (ref)		1.0 (ref)	
Yes	0.9	0.23	**1.2**	**0.02**	0.6	0.17	1.2	0.13
**3. Comorbidities**								
Hypertension								
No	1.0 (ref)		1.0 (ref)		1.0 (ref)		1.0 (ref)	
Yes	1.2	0.14	1.0	0.88	1.1	0.86	1.2	0.16
Diabetic mellitus								
No	1.0 (ref)		1.0 (ref)		1.0 (ref)		1.0 (ref)	
Yes	**1.3**	**0.05**	**1.4**	**<0.01**	**2.7**	**0.04**	**1.4**	**0.02**

Table 7 presents the effect of hypertension and diabetes mellitus (DM) on the risk of cataract. RRs with CIs are presented separately for the DM group (RR = 1.7, 95% CI, 1.4-2.2), for the hypertension group (RR = 1.2, 95% CI, 1.1-1.3), and for both groups combined (RR = 2.0, 95% CI, 1.6-2.5); subjects without DM or hypertension were the reference group.

**Table 7:** Modification of the effect of diabetes mellitus on risk of developing cataracts by hypertension

	**Diabetes Mellitus (DM)**				**RR (95% CI); p for with versus without DM within strata of hypertension**
	**No**		**Yes**		
**Hypertension**	**%**	**RR (95% CI)**	**%**	**RR (95% CI)**	
No	2852/7502 (38%)	1.0	345/531 (65%)	1.7 (1.4–2.2) p < 0.01	1.7 (1.4–2.2) p < 0.01
Yes	1757/2735 (64%)	1.2 (1.1–1.3) p < 0.01	565/736 (77%)	2.0 (1.6–2.5) p < 0.01	1.1 (0.9–1.5) p = 0.37

Measure of effect modification on additive scale: RERI (95% CI) = 0.1(−0.5 ~ 0.6); p = 0.85.

Measure of effect modification on multiplicative scale: ratio of RRs (95% CI) = 1.0 (0.7 ~ 1.3); p = 0.78.

RRs are adjusted for age, sex, household monthly income, and education.

RR: relative risk, RERI; relative excess risk due to interaction, CI; confidence interval.

## Discussion

This study assessed results from a national health survey to provide epidemiologic data on the prevalence of cataract among Koreans aged 40 years and older. In doing so, we found a prevalence of 40.1% for any cataract or cataract surgery. Additionally, our study showed that the frequencies of cataract between both genders were similar, and that hypertension and DM could be modifiable risk factors.

Much of the literature does not report the prevalence of each subtype of cataract. Among the articles that have reported on the prevalence of each subtype, the studied age groups vary, some of which included those over the age of 40 [20-22,30] or even those over the age of 50 [19], 60 [31], or 65 [32]. Moreover, the grading systems utilized for the assessment of cataract also vary; LOCS III was used in only some of the studies [20,22,30-32]. The focus of the present study was not to compare the prevalences of cataract reported in the literature, but rather to assess the prevalence in the Korean population and investigate potential risk factors.

### Prevalence of cataracts in Asian Countries

The prevalence of cataracts in Asian countries, including Singapore [20,22], Taiwan [32], Japan [19], China [21], Myanmar [30], India [31] and Pakistan [33] ranged from 20% [33] to 63% [31]. For the subtypes of cataracts in Asian countries, cortical cataract prevalence ranged from 7.1% [31] to 23.9% [22], nuclear cataract prevalence ranged from 22.6% [22] to 50.3% [21], and PSCO prevalence ranged from 4.3% [21] to 18.7% [31]. Compared to prevalence values reported in previous studies, those found in our study for pure cortical, nuclear, or PSCO types were lower, whereas more cataracts were classified as the mixed type, possibly because we used a more strict classification system for evaluating the risk factors associated with the pure subtype. In our study, the prevalence of the pure nuclear type was more than twice that of the pure cortical type. This did not surprise us because many previous studies, including studies from Taiwan [32], China [21], Myanmar [30] and India [31] reported a higher prevalence of the nuclear type than the cortical type. Furthermore, the Indian study [31] compared its northern population to its southern population and concluded that the northern population had a higher prevalence of the nuclear type cataract (42.2% versus 34.5%), which was probably due to environmental factors such as climate and/or ultraviolet exposure. Some of these studies discussed prevalence differences between populations, despite some debate as to whether differences result from environmental or racial/genetic differences [19,34].

### Age

Of all significant factors from this study, age was the most significant risk factor for cataracts, as in previous studies [17,21,30,31,35]. With increased age, one is more likely to suffer from cumulative exposure to numerous risk factors, especially environmental factors, such as either longer duration of radiation or oxidative damage [36]. We found it interesting that our odds ratio for the mixed type was significantly higher than that for either the cortical or the nuclear type (Table 6). This result implies that a patient with a mixed type cataract is most likely to be an older person, as compared to patients with pure cortical, nuclear, or PSCO cataracts. As this study is a cross-sectional study, we can show neither its time-sequence nor any causal relationships between age and mixed type cataracts. The mixed type might have resulted from multiple pathogenesis from exposure to multiple risk factors; therefore, the older person, with presumably more exposure to various pathogenesis and risk factors, would be linked to the mixed type cataract.

### Socioeconomic status and educational status

Others have investigated socioeconomic status and educational status risk factors for cataracts, with various results [2,37]. In our study, individuals with lower incomes were associated with pure cortical and mixed type cataracts, whereas lower education status was associated with pure cortical and nuclear type cataracts. Although educational status could have a dependent relationship with socioeconomic status, our study actually shows it to be an independent risk factor for cataracts (Table 5). Socioeconomic status and educational status are general ways of living that could produce risk factors that may not yet be described. Here, we can only suggest the possibility of more risk factors associated with socioeconomic status and/or educational status.

### DM and hypertension

Our study reconfirms the positive relationship that hypertension and DM have with cataract prevalence [1,5,38-40]. Many studies show DM to be related to cortical, nuclear, posterior subcapsular, and mixed type cataracts [4,5,41,42]. Our study also showed prominent odds ratios for all cataract subgroups. The overall odds ratio for DM and any type of cataract was the second highest (aOR = 1.6; 95% CI, 1.3–1.9), and the odds ratio for the posterior subcapsular type was the highest in the subgroup analysis (aOR = 2.7; 95% CI, 1.1–6.9).

According to Strengthening the Reporting of Observational Studies in Epidemiology (STROBE), we should consider hypertension and DM effect modifications. Because cataract prevalence is not rare, we were confident we could perform additional analyses of relative risks (and their 95% CIs) to avoid exaggerated interactions in Table 7. When we considered hypertension and DM, participants who had only DM were more likely to have cataracts than those with only hypertension, and those with both hypertension and DM were two times more likely to have cataracts than those with neither hypertension nor DM (Table 7).

Hypertension has been of interest as a risk factor in previous studies. Cross-sectional analysis on an initial Beaver Dam Eye cohort showed a correlation between hypertension and posterior subcapsular type [43], whereas a Blue Mountain Eye cohort (from 10-year incidence data) had a relationship with a high rate of cataract surgery [44]. A recent cross-sectional study from the Los Angeles Latino Eye Study showed a relationship between hypertension and both posterior subcapsular and mixed type cataracts [45]. In our study, we were only able to show a relationship between hypertension and the “overall” cataract population.

### Hypercholesterolemia

Although some studies have shown that statins have a protective effect against cataract development, not only because of their cholesterol-lowering effects but also possibly due to anti-oxidative and/or anti-inflammatory effects [46,47], dyslipidemia might still be a risk factor for cataract development. In our study, we included hyper-Low-Density-Lipoproteinemia (hyper-LDL), hypo-High Density Lipoproteinemia (hypo-HDL), and hypertriglycemia (hyper-TG) as separate variables in our initial univariate logistic analysis (Table 3), and we later showed that hypercholesterolemia was the only independent risk factor for cataracts after multivariate logistic analysis (aOR = 1.2; 95% CI, 1.0–1.3). Further tests on the association between nutrition and cataract development might reveal more information.

### Metabolic syndrome

Abdominal obesity is associated with insulin resistance on peripheral glucose and fatty acid utilization, often leading to co-occurrence of metabolic risk factors for type 2 DM, dyslipidemia, hypertension, and cardiovascular diseases [48,49]. These studies evaluated metabolic syndrome as an independent risk factor for cataracts, but when more components of the metabolic syndrome were included in a prospective cohort study, more risk was reported [50]. In our study, metabolic syndrome was an independent risk factor for the pure nuclear cataract type (Table 4).

There are some limitations to this study since the KNHANES and its ophthalmologic examinations aimed to investigate various health issues, limited resources were allocated for investigating cataract and subtypes. First, iridodilators could not be used, which could have influenced the detection and classification of the cataract, even though the examiners maximized the pupil diameter with the illuminator and slit lamp settings described in the Methods section, this could have been an underestimation of cataract prevalence. Misclassification and under estimation were possible, especially for the cortical type of cataract when it occurs in the peripheral cortex in a circumferential or in an arcuate pattern. Second, the cut value of opalescence based on LOCS III score was not included, which could induce individual variation between ophthalmologists; however, while grading and classifying by a few ophthalmologists may increase the accuracy of the result, a systemic error could also increase. This could reflect the generally accepted definition of cataract from a number of ophthalmologists. Third, the ophthalmologic exam for this study was done with a slit lamp without a permanent photographic record, which could have restricted the reviewing or assessment of inter-observer reliability for the classification. Despite these limitations, we believe our study adequately identified risk factors most associated with cataract development, particularly at the national level.

## Conclusions

We were able to show that socioeconomic disparities do exist in cataract development, and improved control of blood pressure, sugar, cholesterol and the factors associated with metabolic syndrome may help to reduce the risk of cataract development. For individuals who have both hypertension and DM, the risk of developing cataracts was twice than that of the individuals without. Therefore, we recommend more targeted efforts to reduce such risks in this group.

## Competing interests

The authors have no proprietary or commercial interest in any materials discussed in this article.

## Authors’ contributions

THTR and MK drafted the manuscript. THTR and WCK carried out the statistical analysis. WCK interpreted the data. TK and EKK prepare and review of the manuscript. EKK participated in study design. This survey was conducted by Epidemiologic Survey Committee of the Korean Ophthalmologic Society. All authors read and approved the final manuscript.

## Pre-publication history

The pre-publication history for this paper can be accessed here:

http://www.biomedcentral.com/1471-2415/14/4/prepub
